# Alizarin and Chrysazin Inhibit Biofilm and Hyphal Formation by *Candida albicans*

**DOI:** 10.3389/fcimb.2017.00447

**Published:** 2017-10-16

**Authors:** Ranjith Kumar Manoharan, Jin-Hyung Lee, Yong-Guy Kim, Jintae Lee

**Affiliations:** School of Chemical Engineering, Yeungnam University, Gyeongsan, South Korea

**Keywords:** *C. albicans*, anthraquinone, alizarin, chrysazin, biofilm formation, hyphal formation

## Abstract

*Candida albicans* is one of the most common pathogen causes fungal infections. This opportunistic pathogen can form biofilms comprised of yeast, hyphae and pseudo hyphal elements, and the hyphal form *C. albicans* considered as probable virulence factor. We investigated the antibiofilm activities of 13 quinones and anthraquinones related compounds against *C. albicans* biofilms by using crystal violet and 2,3-bis (2-Methoxy-4-Nitro-5-Sulfo-phenyl)-2H-Tetrazolium-5-Carboxanilide (XTT) reduction assays to assess inhibitions of biofilm growth. Morphological changes in biofilms and biofilm thicknesses were determined by scanning electron microscopy and confocal laser scanning microscopy, respectively. It was found alizarin (1,2-dihydroxyanthraquinone) and chrysazin (1,8-dihydroxyanthraquinone) suppressed *C. albicans* biofilm formation. Interestingly, alizarin and chrysazin at only 2 μg/ml effectively inhibited hyphal formation and prolonged the survival of *C. albicans* infected *Caenorhabditis elegans*, thus showing a distinct antivirulent potential. A structural activity relationship study of alizarin and 6 other anthraquinones showed the presence of a hydroxyl group at C-1 position which is important for antibiofilm and antifilamentation activities. Transcriptomic analyses revealed that alizarin downregulated the expression of several hypha-specific and biofilm related genes (*ALS3, ECE1, ECE2*, and *RBT1*). Furthermore, unlike the commercial antifungal drug fluconazole, no acute toxic effect was observed when uninfected nematodes were exposed to alizarin at concentrations up to 1 mg/ml. The results of this study indicate alizarin suppresses the virulence of *C. albicans in vivo* which suggests alizarin may be considered as a potential candidate for further investigations to develop antifungal agent against fungal pathogen *in vivo*.

## Introduction

*Candida albicans* is an opportunistic fungal pathogen and the cause of systemic infections predominantly in immunocompromised individuals and in patients with an implanted device, such as, a catheter, cardiac pacemaker, or heart valve (Ramage et al., 2005; Sardi et al., 2013). *C. albicans* can grow as oval budding yeasts, as continuous septate hyphae, or as pseudohyphae, and all three of these morphological forms are usually observed in infected tissues. *C. albicans* can easily colonizes on medical devices such as gastrointestinal tract and intravascular catheters, artificial heart valves, and contact lenses and cause infections with high mortality rates (Sardi et al., 2013). Identified virulence factors of *Candida* infection include initial adhesion and the ability to form surface biofilms, the latter of which causes yeast cell transition to their hyphal form (Rajasekharan et al., 2015; Carradori et al., 2016). Accordingly, the suppressions of biofilm formation and hyphal transition are considered as effective strategies for countering *Candida* virulence and pathogenesis (Gauwerky et al., 2009). The dimorphologic states (yeast and hypha) of *C. albicans* cells and the inhibition of phenotype switching between yeast and hyphae presents a possible means of developing antifungal agents. Furthermore, the effectivenesses of available antifungals are limited by the development of resistant *Candida* biofilms and by their toxicities (Taff et al., 2013; Sandai et al., 2016). Thus, there is an urgent need for new antifungals that are effective against *Candida* biofilms.

Several potential molecules like linalool (Souza et al., 2016; Manoharan et al., 2017a), geraniol (Cardoso et al., 2016), nerolidol (Curvelo et al., 2014), sophorolipid (Haque et al., 2016), gymnemic acid (Vediyappan et al., 2013), and phenazines (Morales et al., 2013) have been reported to inhibit biofilm formation by *Candida*, and a few small molecules, such as, shearinine, clozapine, buhytrin A, and α-longipinene, have been reported to inhibit *C. albicans* yeast to hypha transition (Grald et al., 2012; Pierce et al., 2015; Reen et al., 2016; Manoharan et al., 2017a). Importantly, compounds that inhibit biofilm formation and hyphal growth without affecting growth or planktonic cell viability, which minimize resistance, might be useful antibiofilm agents. Few researchers have reported several anthraquinones, such as, purpurin, emodin, chyrsophanol, rubiadin, and rhein, with antifungal and antibiofilm activities against *C. albicans* (Xiang et al., 2008; Kang et al., 2010; Marioni et al., 2016; Janeczko et al., 2017). Previously, we investigated the antibiofilm activities of 560 phytochemicals against *Staphylococcus aureus*. Of these several anthraquinone derivatives had showed biofilm inhibition against *S. aureus* (Lee et al., 2016). Hence, the present study was designed to investigate the effect of anthraquinone derivatives against *C. albicans* virulence.

In the present study, we report the abilities of anthraquinone derivatives to inhibit biofilm formation without affecting the planktonic growth of *C. albicans* using crystal violet and XTT [2,3-bis(2-methoxy-4-nitro-5-sulfo-phenyl)-2H-tetrazolium- 5-carboxanilide] reduction assays. Cell morphology and phenotypic switching of *C. albicans* cells were observed by scanning electron microscopy (SEM) and biofilm thicknesses was measured by confocal laser scanning microscopy (CLSM). In addition, potential compounds were evaluated with respect to hyphal inhibition and anti-biofilm efficacy using a *Caenorhabditis elegans* (a nematode) model.

## Materials and methods

### Strains, cultivation, chemicals, and minimum inhibitory concentrations

The standard *C. albicans* strains DAY185, ATCC10231, ATCC18804, and ATCC24433 used in this study were obtained from the Korean Culture Center of Microorganisms (http://www.kccm.or.kr/). Streaking and subculturing of *C. albicans* strains were performed using potato dextrose agar (PDA) or potato dextrose broth (PDB), unless otherwise specified. *C. albicans* strain was preserved at −80°C in 1 ml of PDB supplemented with 30% glycerol stock, and when needed, streaked on PDA plates. Plates were incubated for 48 h at 37°C, and a fresh single colony was then inoculated into 25 ml of PDB and cultured overnight at 37°C. The strain *S. aureus* 6538 was maintained and cultured in LB medium. All 13 compounds tested, namely, alizarin, alizarin red, anthraflavic acid, anthraquinone, chrysazin, (+)-catechin, (+)-catechin hydrate, emodin, 1-hydroxyanthra-9,10-quinone, hydroquinone, purpurin, pyrocatechol, and quinalizarin were purchased from Sigma-Aldrich (St. Louis, USA) and dissolved in dimethyl sulfoxide (DMSO), which did not exceed 0.1% (vol/vol) in any experiment. To determine cell growths, turbidities were measured at 620 nm using a spectrophotometer (UV-160, Shimadzu, Japan). Minimum inhibitory concentrations (MICs) were determined using the Clinical Laboratory Standards Institute (CLSI) broth dilution method with slight modification (Alastruey-Izquierdo et al., 2015), using 96-well polystyrene plates (SPL Life Sciences, Korea). *C. albicans* cells were cultured overnight in PDB medium and diluted to reach a final concentration of 10^5^ CFU/mL and added to the wells in the presence of varying concentrations (w/v) of tested compounds at 24 h at 37°C. MIC was defined as the lowest concentration that inhibited microbial growth by at least 80%, as assessed by spectrophotometry (620 nm) and colony counting. MICs of tested compounds are expressed as percentages (v/v or w/v).

### Assays of biofilm inhibition and biofilm dispersal

*Candida* biofilms were prepared on 96-well polystyrene plates, as previously reported (Lee et al., 2011). Briefly, overnight cultures of *C. albicans* strains were inoculated into PDB (total volume 300 μl) at an initial turbidity of 0.1 at 600 nm and cultured with or without test compounds at varying concentrations for 24 h without shaking at 37°C. To perform biofilm inhibition assay in mixed cultures, overnight cultures of *C. albicans* and *S. aureus* strains were equally inoculated into medium containing PDB and LB (final volume 300 μl) at an initial turbidity of 0.1 at 600 nm and cultured as mentioned above.

To investigate the effects of the anthraquinones alizarin and chrysazin on biofilm disruption, *C. albicans* biofilms were prepared on 96-well polystyrene plates for 24 h at 37°C, as described above. Briefly, wells were washed twice with PBS and fresh PDB medium (300 μl) containing different concentrations of compounds were added to plates, which were then further incubated for 24 h at 37°C. Biofilm formation was quantified after washing three times with H_2_O (to remove all non-adherent cells), staining with crystal violet for 20 min, rinsing three times with H_2_O, and extracting the crystal violet with 95% ethanol. Absorbance was measured at 570 nm, and results are presented as the averages of at least six replicates.

### Biofilm metabolic activity—XTT reduction assay

A colorimetric XTT [2,3-bis(2-methoxy-4-nitro-5-sulfophenyl)-2H-tetrazolium-5-carboxanilide sodium salt] reduction assay was performed using established procedures (Ramage et al., 2001; Nett et al., 2011). Briefly, overnight cultures of *C. albicans* strains were inoculated into PDB (total volume 300 μl) at an initial turbidity of 0.1 at 600 nm and cultured with or without alizarin or chrysazin at different concentrations for 24 h without shaking at 37°C. A XTT reduction kit (Sigma-Aldrich, St. Louis, USA) was used to measure the metabolic activities of biofilm cells. XTT and menadione solutions were mixed at 20:1 (v/v) immediately prior to the assay. PBS was then added to XTT-menadione solution (3.76:1 v/v) and 200 μl of this mix was added to each well containing pre-washed biofilms, and incubated in the dark for 3 h at 37°C. The colored supernatant (100 μl) so obtained was transferred to new microtiter plates, and optical densities were measured at 450 nm. Similarly, planktonic cell viability was measured by using culture supernatants.

### Assay of *C. albicans* hyphal development in liquid media

Overnight cultures of *C. albicans* DAY 185 were inoculated into the hyphae-inducing media RPMI-1640 medium with or without alizarin or chrysazin (both at 2 μg/ml) for 24 h with shaking at 37°C. Aliquots of fungal cells were harvested at different times (0, 6, or 24 h) and visualized under bright field using the iRiS™ Digital Cell Imaging System (Logos Bio Systems, Korea).

### Confocal laser scanning microscopy of biofilm formation

*C. albicans* cells were cultured in 96-well polystyrene plates (SPL Life Sciences, Korea) without shaking in the absence or presence of alizarin or chrysazin. Planktonic cells were then removed by washing with PBS buffer three times. *C. albicans* cells were stained with carboxyfluorescein diacetate succinimidyl ester (a minimally fluorescent lipophile; Catalog #: C34554, Invitrogen, Molecular Probes, Inc, Eugene, USA; Weston and Parish, 1990), which becomes highly fluorescent when it loses its acetyl groups due to the action of esterases in cells. Biofilms at the bottom of plate was visualized using an (a 488 nm) Ar laser (emission wavelength 500–550 nm) under a confocal laser microscope (Nikon Eclipse Ti, Tokyo) equipped with a 20 × objective (Kim et al., 2012). Color confocal images were constructed using NIS-Elements C version 3.2 (Nikon eclipse). For each experiment, at least 10 random positions in two independent cultures were examined.

### Observations of hyphae by scanning electron microscopy (SEM)

SEM was used to observe hyphal formation, as previously described (Lee et al., 2014). Briefly, a nylon membrane was cut into 0.5 × 0.5 cm pieces and one piece was placed per well in 96-well plates containing 200 μL cells/well of turbidity 0.05 at 600 nm. Cells were incubated in the presence or absence (untreated control) of alizarin or chrysazin at 37°C for 24 h without shaking. Cells were then fixed with glutaraldehyde (2.5%) and formaldehyde (2%) for 24 h, and post fixed in osmium tetroxide and dehydrated in a series of ethanol solutions (50, 70, 80, 90, 95, and 100%), and isoamyl acetate. After critical-point drying, cells fixed onto nylon membranes were examined under a S-4200 scanning electron microscope (Hitachi, Japan) at a voltage of 15 kV and magnifications ranging from × 2,000 to × 10,000.

### RNA isolation and quantitative real-time PCR (qRT-PCR)

For transcriptional analysis, *C. albicans* was inoculated into 25 ml of PDB broth in 250 ml shake flasks at a starting OD_600_ of 0.1, and then cultured at 37°C for 4 h with agitation (250 rpm) in the presence or absence of alizarin or chrysazin (2 μg/ml). RNase inhibitor (RNAlater, Ambion, TX, USA) was added to prevent RNA degradation. Total RNA was isolated using a hot acidic phenol method (Amin-ul Mannan et al., 2009) and further proceed to clean up this RNA with Qiagen RNeasy mini Kit (Valencia, CA, USA). qRT-PCR was used to determine the transcription levels of hypha- and biofilm-related genes [*HYR1, EFG1, ECE1, ECE2, ALS1, ALS3* (or called *HWP1*)*, EED1*, and *RBT1*] in *C. albicans* treated or not with alizarin or chrysazin. Gene specific primers were used and *RDN18* was used as housekeeping controls (Supplementary Table 1). The qRT-PCR method used has been previously described (Lee et al., 2011). qRT-PCR was performed using a SYBR Green master mix (Applied Biosystems, Foster City, USA) and an ABI StepOne Real-Time PCR System (Applied Biosystems) on two independent cultures.

### Candida infection in the *Caenorhabditis elegans* model

To investigate the effects of alizarin or chrysazin or purpurin on the virulence of *C. albicans*, the nematode *C. elegans* wild type strain N2 Bristol CF512 *fer-15(b26); fem-1(hc17)* (Murphy et al., 2003; Oh et al., 2012) with loss of sex-determining protein Fem-1 and age determining protein Fer-15 was infected with *C. albicans* as previously described (Manoharan et al., 2017a). Briefly, a freshly prepared overnight *C. albicans* culture (100 μl) was spread onto a lawn on PDA plates and incubated for 48 h at 37°C. Synchronized adult were then allowed to feed on the *C. albicans* yeast lawn for 4 h at 25°C when worms were collected and washed three times with sterile M9 buffer. Approximately 10 worms were then pipetted into single wells of 96-well plates containing PDB medium and treated with solutions (300 μl) of investigated compounds at final concentrations ranging from 0.2 to 2 μg/ml. Controls were treated with medium alone. For toxicity assays, 10 non-infected worms were pipetted into single wells of a 96-well dish containing M9 buffer and solutions of the compounds (300 μl) were added to final concentrations of 1 or 2 mg/ml. Plates were then incubated at 25°C for 4 days with gentle shaking. Three independent experiments were conducted in triplicate. Results are expressed as percentages of alive or dead worms by gently touching them with a platinum wire after 4 days of incubation, and results were obtained using an iRiS™ Digital Cell Imaging System (Logos Bio Systems, Korea).

### Statistical analysis

In all cases, at least two independent experiments were conducted, and results are expressed as means ± standard deviations. The student's *t*-test was used to determine the significances of differences between treated and non-treated samples. Statistical significance was accepted for *p* < 0.05, and in the figures significant changes are indicated by asterisks.

## Results

### Effects of anthraquinone and quinone related compounds on *C. albicans* biofilm formation

**Figure 1:**
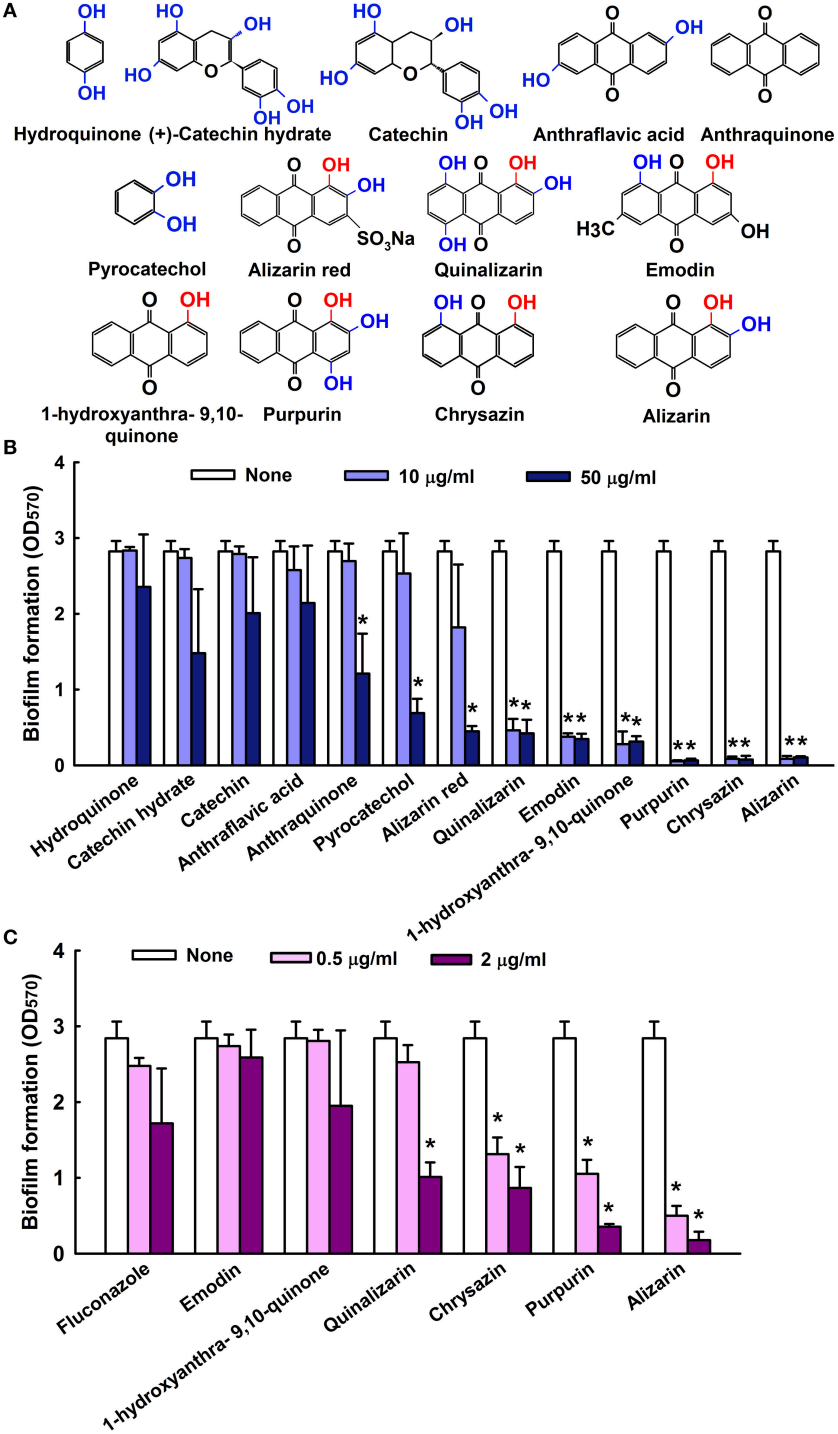
Inhibition of biofilm formation by quinone and anthraquinone related compounds. Chemical structures are shown. Hydroxyl groups are shown in blue and hydroxyl groups at para positions are in red **(A)**. The antibiofilm activities of quinones and anthraquinones related compounds against *C. albicans* were determined after culturing for 24 h **(B)**. Antibiofilm formation by selected compounds at low doses **(C)** none indicates non- treated samples. Two independent experiments were conducted (6 wells per sample); error bars indicate standard deviations. ^*^p < 0.05 vs. non-treated controls.

**Figure 2:**
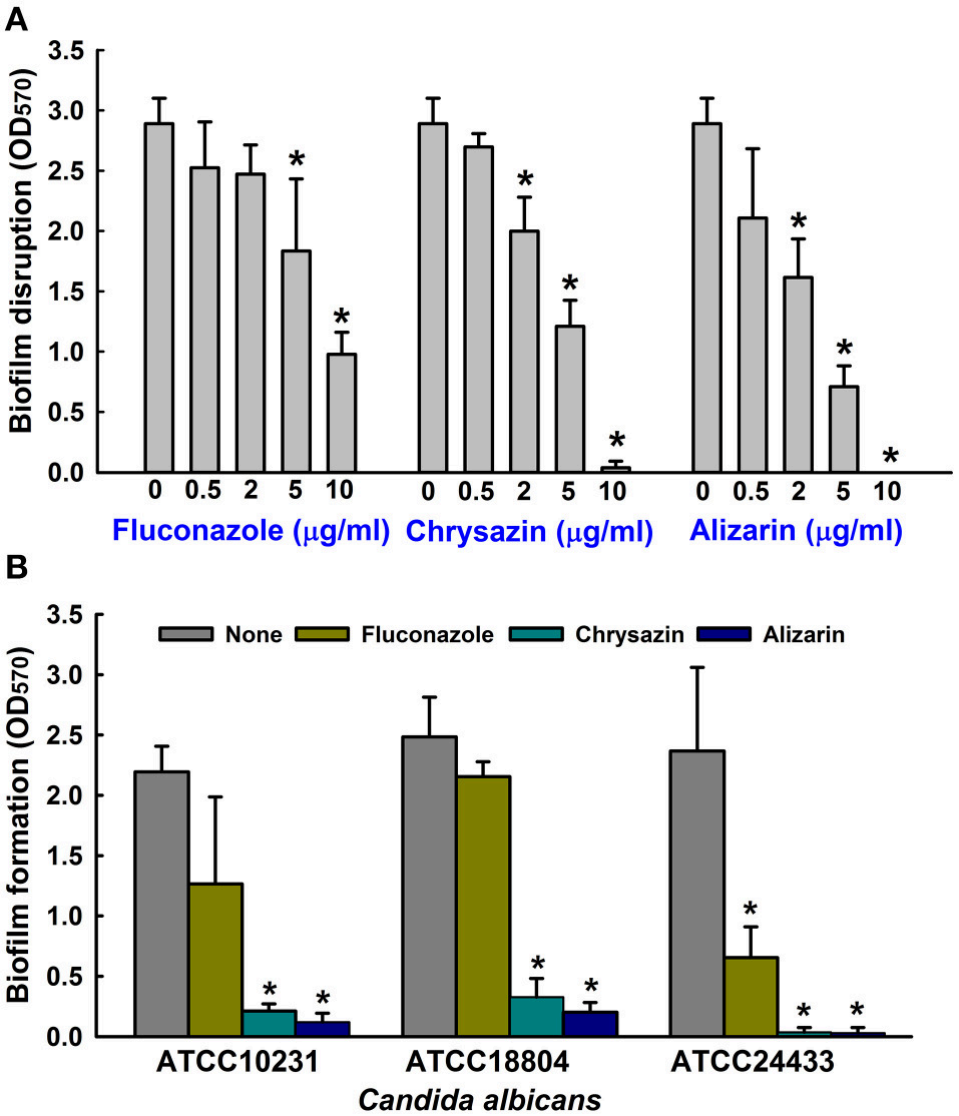
Antibiofilm activities of alizarin and chrysazin against *C. albicans*. Preformed biofilms of *C. albicans* were treated with alizarin, chrysazin, or fluconazole for 24 h **(A)**. At least two independent experiments were conducted (6 wells per sample). The antibiofilm activities of alizarin, chrysazin and fluconazole at 2 μg/ml were investigated by culturing different *C. albicans* strains for 24 h in 96-well polystyrene plates **(B)**. At least two independent experiments were conducted (6 wells per sample). Error bars indicate standard deviations. ^*^p < 0.05 vs. non-treated controls.

The antibiofilm efficacies of the 13 compounds (Figure 1A) tested were examined using *C. albicans* on 96-well polystyrene plates. Of these compounds, the anthraquinones alizarin, chrysazin, purpurin, 1-hydroxyanthra-9,10-quinone, emodin, and quinalizarin were found to be effective at 10 μg/ml against *C. albicans* biofilms without affecting planktonic cell growth (Figure 1B). Nevertheless, anthraquinone, pyrocatechol, and alizarin red showed significant biofilm inhibition at 50 μg/ml (Figure 1B). Further experimentation showed that chrysazin, purpurin, and alizarin significantly reduced biofilm formation by 50, 57, and 82%, respectively, even at 0.5 μg/ml (Figure 1C). Further increases in the concentrations of alizarin, chrysazin, and fluconazole significantly exacerbated biofilm disruption (Figure 2A). In addition, alizarin and chrysazin at 2 μg/ml dramatically reduced biofilm formation by three other *C. albicans* strains (ATCC10231, ATCC18804, ATCC24433) by ≥90%. While fluconazole significantly reduced biofilm formation by *C. albicans* strains ATCC10231 and ATCC24433 by 43 and 72%, respectively at 2 μg/ml (Figure 2B). Our data suggest that alizarin and chrysazin are more effective to inhibit biofilm formation by several *C. albicans* strains than commercial drug fluconazole. Interestingly, alizarin, chrysazin, and purpurin had significantly reduced mixed biofilm formation by *C. albicans* and *S. aureus* at 2 and 10 μg/ml, while fluconazole had lower activity on mixed biofilms at 10 μg/ml when compared to anthraquinone derivatives (Supplementary Figure 1). Since purpurin has been reported as antibiofilm and antihyphal agent against *C. albicans* (Tsang et al., 2012), our present study was designed and focused only on alizarin and other anthraquinone related compounds for their antihyphal activities.

### Effects of alizarin and chrysazin on *C. albicans* metabolic activity

**Figure 3:**
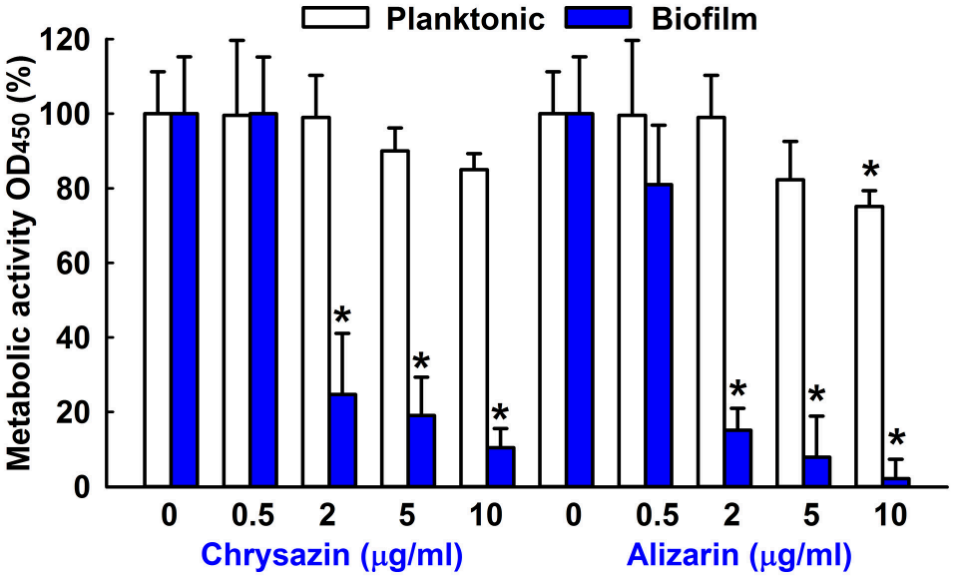
Metabolic activity of alizarin and chrysazin against *C. albicans*. The metabolic activities of planktonic cells and biofilms of *C. albicans* were quantified using an XTT assay in the presence of alizarin and chrysazin after incubation for 24 h. Results are presented as mean percent of metabolic activities vs. non-treated controls. Two independent experiments were conducted (6 wells per sample); error bars indicate standard deviations. None indicates non-treated samples. ^*^p < 0.05 vs. non-treated controls.

The effects of alizarin and chrysazin on *C. albicans* biofilms and planktonic cells were quantified using XTT reduction assay and viabilities were calculated by expressing metabolic activities as percentages of non-treated controls. *C. albicans* metabolic activity was significantly reduced (by 80%) by alizarin up to 2 μg/ml. However, chrysazin reduced the metabolic activity of *C. albicans* biofilms by 66% at 2 μg/ml (Figure 3), and alizarin at 10 μg/ml completely removed biofilms (>98%), while it slightly reduced (25%) metabolic activity on planktonic cells.

### Antimicrobial activities of alizarin and chrysazin

The antimicrobial activities of alizarin and chrysazin were investigated by measuring minimum inhibitory concentrations (MIC), which for both compounds were >2,000 μg/ml against *C. albicans*. Notably, this MIC was 1,000-times higher than the concentration (2 μg/ml) required for antibiofilm activity for both compounds. The MIC of all quinone and anthraquinone derivatives are given in Table 1. *C. albicans* DAY185 was found to be resistant to all tested compounds. For example, anthraflavic acid had showed a MIC up to 500 μg/ml, several compounds exhibited MIC ranging from 1,000 to 5,000 μg/ml. These results confirm that biofilm formation by *C. albicans* was effectively reduced by the antibiofilm activities of alizarin and chrysazin and not by their antifungal activities. These results show that alizarin and chrysazin have fungistatic rather than fungicidal effects on *C. albicans*.

**Table 1:** Minimum inhibitory concentration (MIC) of quinone and anthraquinone derivatives against *C. albicans* DAY185.

**Compounds**	**Alizarin**	**Alizarin red**	**Anthraflavic acid**	**Anthraquinone**	**(+)- Catechin**	**(+)- Catechin hydrate**	**Chrysazin**
MIC (mg/ml)	>2	>5	0.5	>5	2.5	>5	>2
**Compounds**	**Emodin**	**1-Hydroxyanthra-9, 10-quinone**	**Hydroquinone**	**Purpurin**	**Pyrocatechol**	**Quinalizarin**	
MIC (mg/ml)	0.5	>5	>5	1.5	2	2.5	

### Alizarin affected *C. albicans* morphology in biofilms

**Figure 4:**
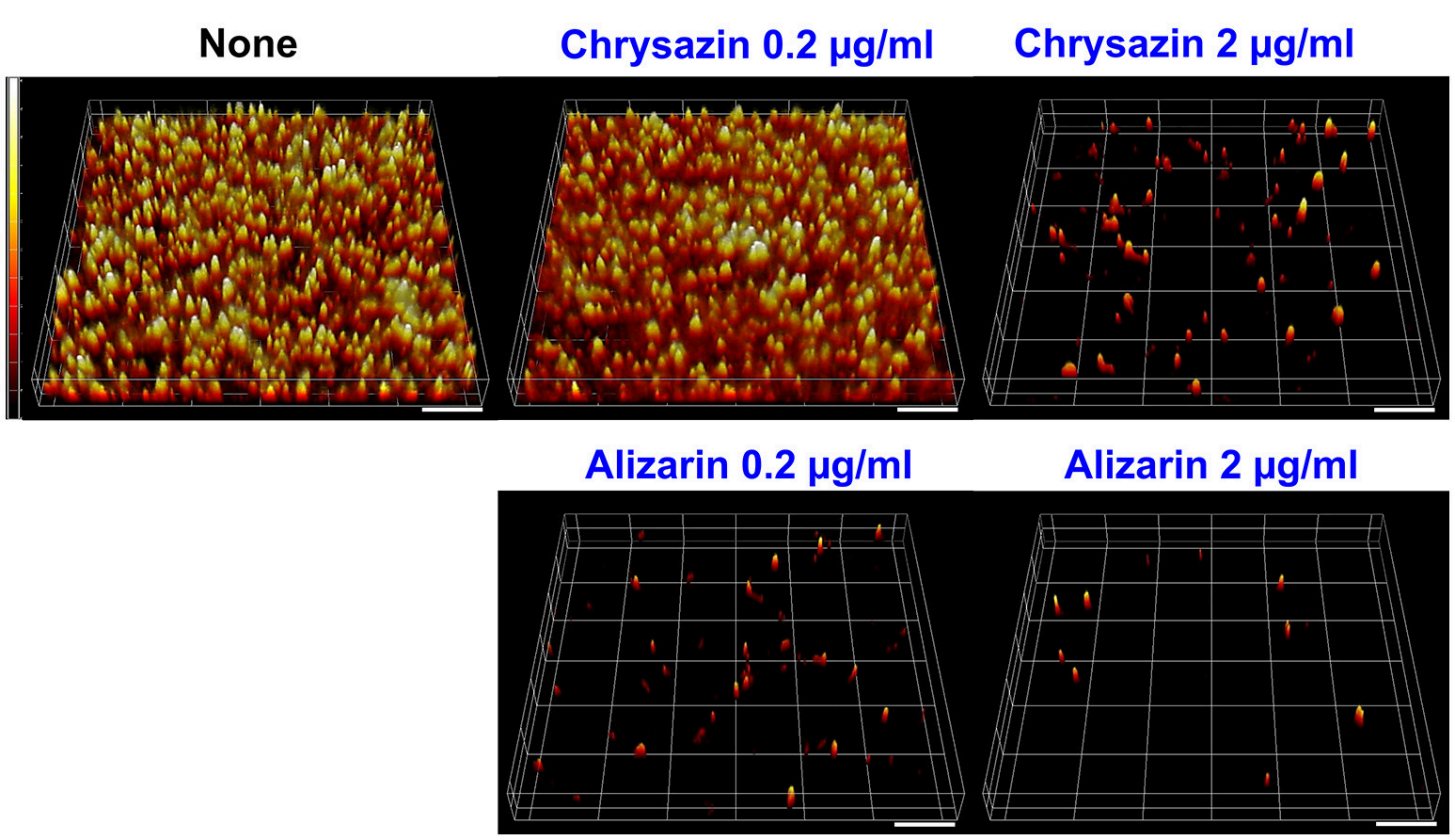
Microscopic observations of the effects of alizarin and chrysazin on biofilms. Biofilm formation by *C. albicans* on polystyrene plates was observed in the presence of alizarin or chrysazin by confocal laser scanning microscopy. Scale bars represent 100 μm.

**Figure 5:**
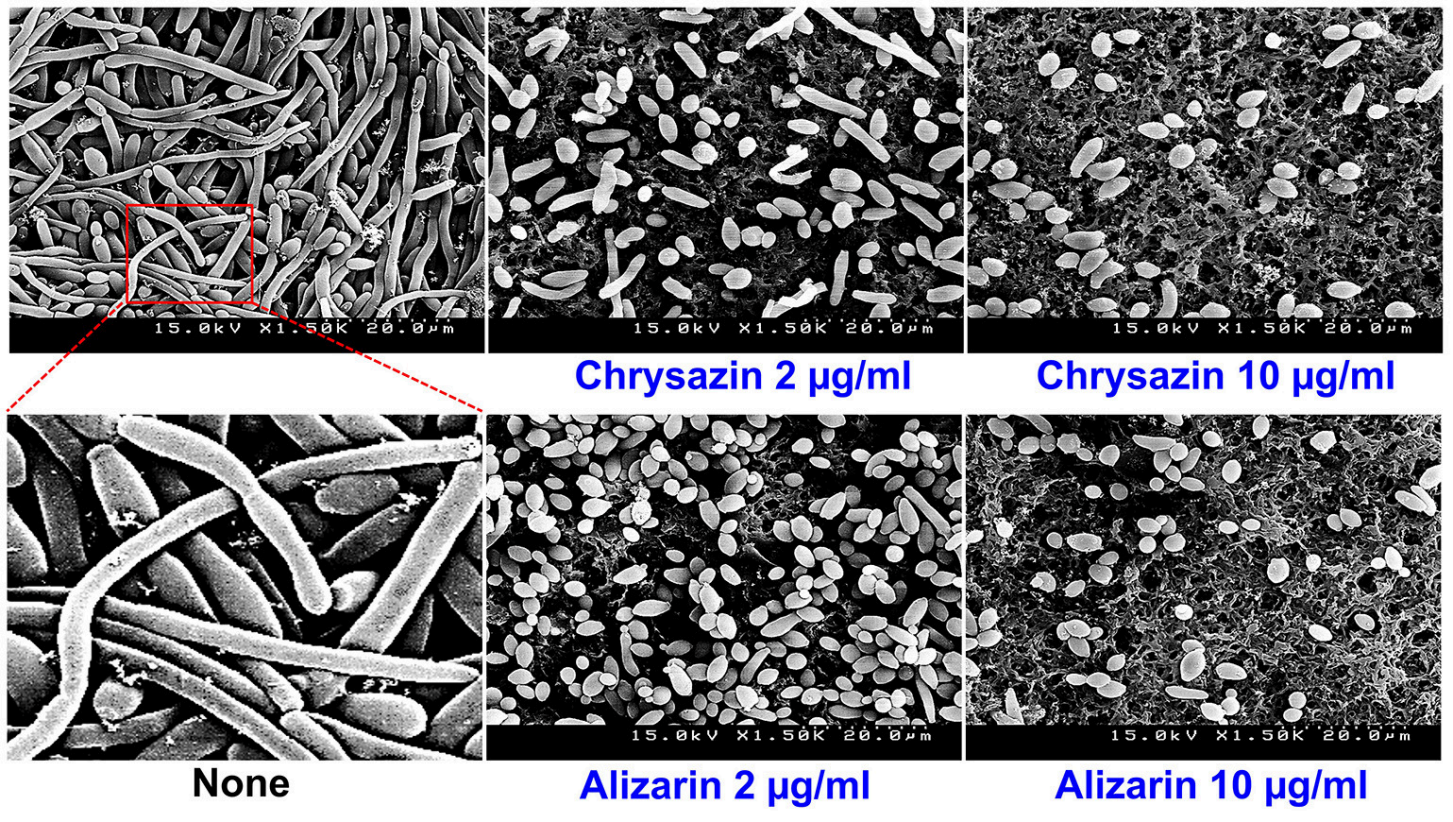
Effects of alizarin and chrysazin on *C. albicans* morphology. Inhibition of hyphal growth was visualized by SEM at a concentration of 2 and 10 μg/ml. The scale bar represents 20 μm. At least two independent experiments were conducted. None indicates non-treated controls.

Confocal laser scanning microscopy (CLSM) images showed *C. albicans* formed dense biofilms in non-treated control samples, but that in the presence alizarin at 0.2 and 2 μg/ml biofilm cellular densities and thicknesses were reduced. While no reduction was observed at a chrysazin concentration of 0.2 μg/ml, major reduction was observed at 2 μg/ml (Figure 4). Scanning electron microscopy (SEM) showed in addition to reducing biofilm metabolic activity, alizarin also inhibited yeast to hyphal transition. As shown in Figure 5, nontreated control biofilms consisted of mixtures of pseudohyphae and hyphae, and few yeast cells. In contrast, alizarin and chrysazin reduces the length of existing hyphae of *C. albicans* and increased yeast form cells as compared to the untreated control (Figure 5). In addition, alizarin and chrysazin both strongly inhibited biofilm development on polystyrene surfaces (Figures 1, 4) and nylon membrane (Figure 5) as well.

### The effects of alizarin and chrysazin on hyphal growth in liquid media

**Figure 6:**
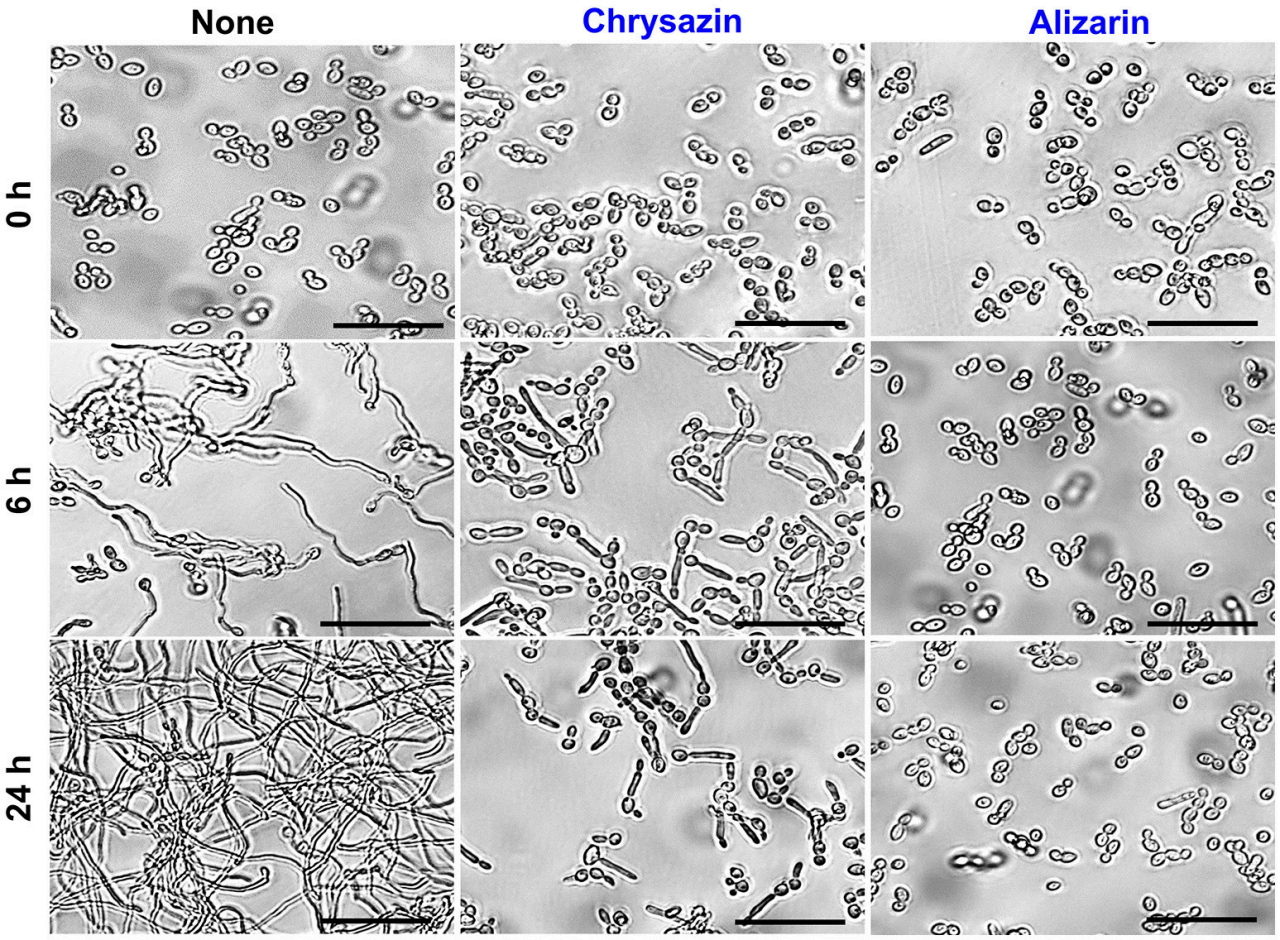
Effects of alizarin and chrysazin on *C. albicans* hyphal growth on liquid media. *C. albicans* was grown in RPMI-1640 medium in the presence of 2 μg/ml alizarin or chrysazin at 37°C for 24 h. Aliquots were withdrawn at different times and photographed using bright field microscope. The scale bar represents 50 μm.

Hyphae inducing RPMI medium was used to examine hyphal inhibition by alizarin and chrysazin. Untreated controls showed massive hyphal growth after 6 h, but in the presence of 2 μg/ml alizarin or chrysazin hyphal growth was not observed. When cultured in RPMI containing alizarin or chrysazin at 2 μg/ml for 24 h, fungal cells grew by budding, whereas controls large hyphal cells were observed (Figure 6) Microscopic observations of alizarin or chrysazin treated fungal cells revealed that in addition to reducing metabolic activity in biofilms, both compounds reduced hyphal lengths and inhibited yeast to hyphae transition.

### Effect of alizarin and chrysazin on the expression of hypha and biofilm related genes

**Figure 7:**
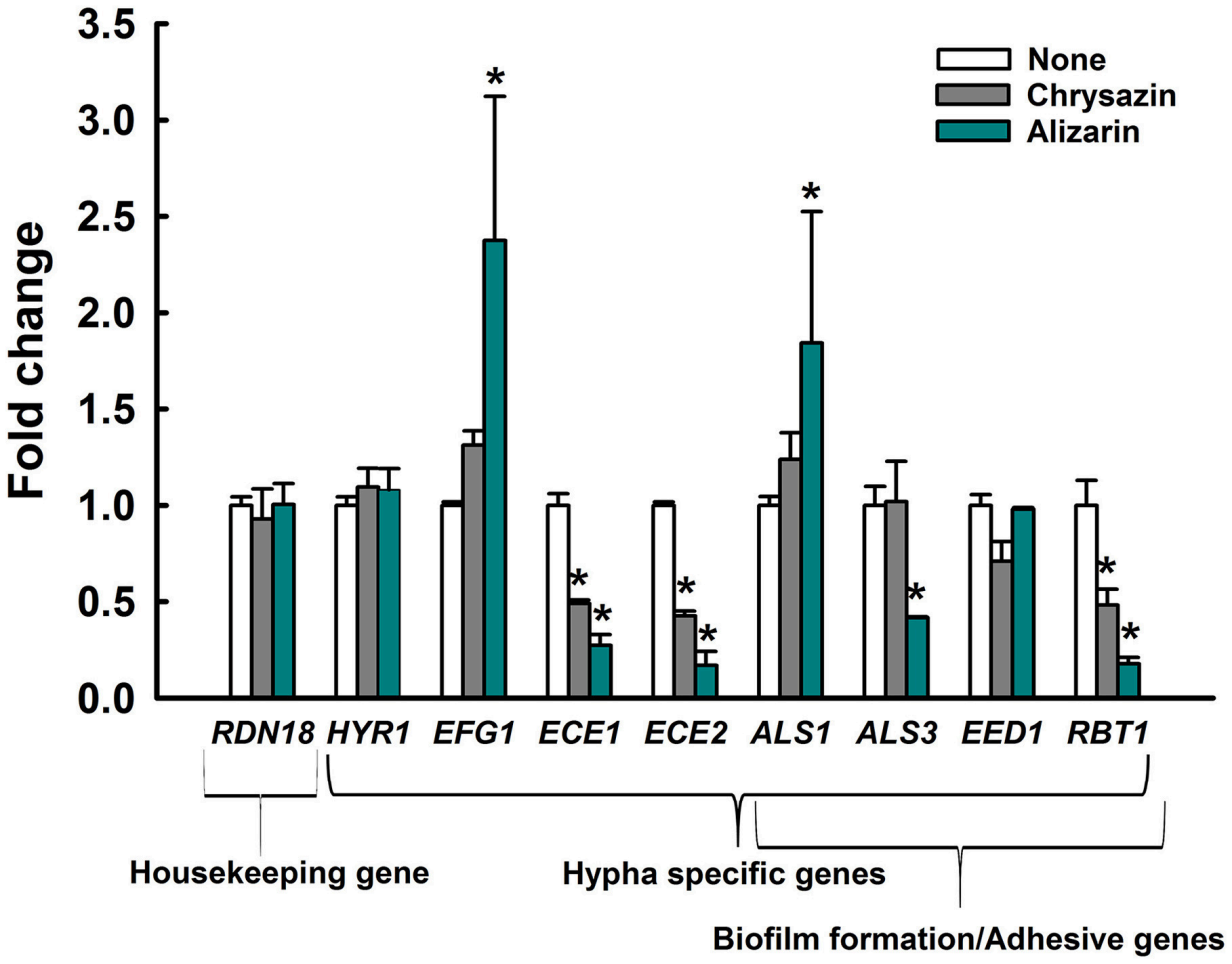
Transcriptional profiles of *C. albicans* cells treated with or without alizarin and chrysazin. *C. albicans* was cultivated with or without alizarin (2 μg/ml) for 4 h. Transcriptional profiles were measured by qRT-PCR. Relative expressions represent transcriptional levels after treatment with alizarin as compared to non-treated controls. Fold changes represents transcription changes in treated *C. albicans* vs. non-treated controls (value of 1.0). The experiment was performed in duplicate. Error bars indicate standard deviations. ^*^p < 0.05 vs. non-treated controls.

Transcriptional levels of the hypha-specific and biofilm related genes in *C. albicans* were quantified by qRT-PCR. Alizarin at 2 μg/ml significantly downregulated the expression of the hypha specific genes *ALS3* (2.4-fold)*, ECE1* (3.7-fold), *ECE2* (6.3-fold), and *RBT1* (5.8-fold) when compared to their respective controls (Figure 7). Similarly, the expression of *ECE1, ECE2*, and *RBT1* were significantly downregulated by 2-, 2.3-, and 2-fold, respectively after chrysazin (2 μg/ml) treatment. Nevertheless, *HYR1* (hypha-specific gene) and *EED1* (adhesive related gene) were not affected after alizarin treatment. Interestingly, *ALS1* which involved in adhesion and biofilm formation was found to be upregulated by alizarin treatment. Similarly, the transcriptional level of hyphae regulatory gene *EFG1* was increased after alizarin treatment. Taken together, qRT-PCR results showed that alizarin significantly altered the expression of some hypha-specific genes and adhesive related genes.

### Inhibition of *Candida* virulence in the nematode *Caenorhabditis elegans*

**Figure 8:**
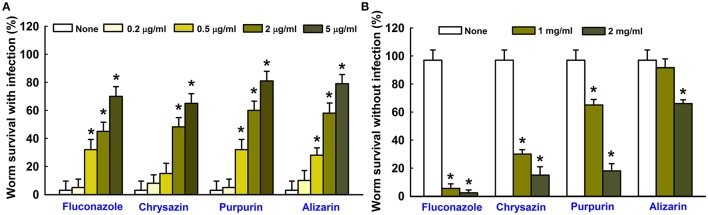
Effects of alizarin or chrysazin or purpurin on *C. albicans* infected *C. elegans*. The bar graph indicates percentage worm survival after exposure of *C. albicans* for 4 days to alizarin or chrysazin or purpurin **(A)**. The toxicities of alizarin or chrysazin or purpurin were studied using non-infected nematodes by determining survival rates after 4 days **(B)**. None indicates non-treated controls. Fluconazole was used as a positive control. Worm survival was determined based on movement. At least two independent experiments were conducted. Error bars indicate standard deviations. ^*^p < 0.05 vs. non-treated controls.

The antibiofilm and antihyphal effects of alizarin and chrysazin were examined in *C. elegans* infected with *C. albicans*. Microscopic observations of infected nematodes revealed that *C. albicans* infection caused 95% fatality in 4 days. However, more than 60% of nematodes survived 4 days in the presence of alizarin (2 μg/ml), >50% survived 4 days in the presence of chrysazin, >60% survived 4 days in the presence of purpurin and <50% survived 4 days in the presence of fluconazole (a commercial antifungal agent; Figure 8A). To examine the toxicities of alizarin and chrysazin, non-infected worms were treated with different concentrations for 4 days. It was found alizarin at concentrations of 1 mg/ml did not affect nematode viability or survival (Figure 8B). However, at this concentration, chrysazin reduced nematodes survival by >60% and purpurin reduced 35% survival rate, whereas only <5% nematodes survived treatment with fluconazole at 1 mg/ml for 4 days. These results show alizarin was as effective as fluconazole at promoting the survival of infected nematodes, but that it was substantially less toxic.

## Discussion

The emergence of multidrug-resistant *Candida* strains and the limited efficacies of commercial antifungal treatments have prompted searches for new antifungals. In the present study, we sought to identify agents that inhibit biofilm formation by *Candida* without reducing its viability. Unlike antibiotics that usually inhibit cell growth, it is important to find biofilm inhibitors that do not affect fungal growth in order to reduce the chance of the development of drug resistance (Iwase et al., 2010). We found that the anthraquinones related compounds namely pyrocatechol, alizarin red, quinalizarin, emodin, 1-hydroxyanthra-9,10-quinone, purpurin, chrysazin, and alizarin inhibited biofilm formation by *C. albicans* in a dose-dependent manner (Figure 1C). Many natural compounds have been tested and found to be effective against various bacterial and yeast virulence traits (De Castro et al., 2013; Hsu et al., 2013; Da Silva et al., 2016). Anthraquinones are usually found in higher plants like *Rubia* species (Usai and Marchetti, 2010; Xu et al., 2014), and several natural and synthetic anthraquinones derivatives have been shown to exhibit antimicrobial and anti-inflammatory activities (Nor et al., 2013; Nam et al., 2017). Furthermore, anthraquinone derivatives like chrysazin have long been employed in medical preparations as laxatives, and most anthraquinones are relatively non-toxic to humans (Sendelbach, 1989; Nam et al., 2017). Previous study found that alizarin, purpurin and other anthraquinone derivatives significantly reduced *S. aureus* biofilms and virulence at a concentration of 2 μg/ml (Lee et al., 2016). The present study shows the anthraquinones alizarin and chrysazin possess potent antibiofilm activity against *C. albicans* strains. Furthermore, since *C. albicans* DAY185 is a fluconazole resistant strain, it would appear that alizarin, which is less toxic than fluconazole, offer a potential means of treatment.

Alizarin is a natural compound derived from the roots of the madder genus, and is used as a red dye in studies on bone growth, calcium deposits in vascular systems, and on gene expression in animal models (Puchtler et al., 1969). Recent study revealed that alizarin had strong *in vitro* activity against human bone tumor cells with lower toxicity to normal cells. In addition to anticancer properties, no tumor promoting or mutagenic activities have been found in human cell lines that suggests the use of alizarin in humans (Takahashi et al., 2002; Fotia et al., 2012). In the present study, alizarin inhibited *C. albicans* biofilm formation by 90% at a concentration of 2 μg/ml, which was a 1,000-fold lower than its MIC for planktonic cells (Figure 1). The presence of aromatic hydroxyls on the anthraquinone ring of alizarin were probably largely responsible for its anti-fungal effect (Tian et al., 2003; Kim et al., 2004), which suggests the presence of OH groups at different positions may have been largely responsible for the different antibiofilm properties observed (Figure 1A). Anthraquinones with an OH group at the para position exhibited highest activity in all assays conducted. For example, purpurin, chrysazin, and alizarin with an OH group in the C1 position (indicated in red in Figure 1A) had showed strong inhibitory activity against *C. albicans* biofilms even at low concentrations (2 μg/ml). In addition to biofilm inhibition, alizarin and chrysazin dose-dependently disrupted matured biofilms (Figure 2A).

Recently, attempts have been made to identify small molecules that modulate hyphal formation by *C. albicans* (Midkiff et al., 2011; Grald et al., 2012; You et al., 2013). Cells that adhere to the surfaces of medical devices could develop biofilm layer, followed by hyphal transition and constitute a significant medical challenge (Kojic and Darouiche, 2004). Fully mature biofilms have a mixture of yeast, hyphae and pseudohyphae morphological forms in the extracellular matrix which suggested that transition of yeast to hyphal form is a directly correlated with development of structured biofilms (Nobile and Mitchell, 2005). Lower concentrations of purpurin (3 μg/ml) and emodin (1.25 μg/ml) have been reported previously for inhibiting the hyphal development against *C. albicans* (Tsang et al., 2012; Janeczko et al., 2017), whereas in the present study, alizarin had showed the same activity at ~2 μg/ml. Furthermore, alizarin and chrysazin at 2 μg/ml significantly disrupted mature biofilms (at 10 μg/ml both completely disrupted), whereas the antifungal fluconazole was less effective against *C. albicans* mature biofilms at this concentration (Figure 2A). In the present study, alizarin and chrysazin completely inhibited hyphal development even at a concentration of 2 μg/ml without affecting cell growth, which may indicate that both alizarin and chrysazin influence the pathway responsible for hyphal development (Figures 5, 6). The data suggest that alizarin and chrysazin suppress *C. albicans* dimorphic switching, which is in agreement with the biofilm development (Figures 4, 5).

The transcriptional levels of several hypha-specific genes were significantly altered in alizarin treated cells. More specifically, *ECE1, ECE2* (or called *HWP1*) and *RBT1* were downregulated after alizarin and chrysazin treatment (Figure 7). Our results were consistent with previous findings that purpurin reduced the transcription levels of hypha-specific genes such as *ALS3, ECE1, HWP1* (Tsang et al., 2012). *ALS3* had been reported as hypha-specific gene that considerably upregulated during initial stages of biofilm formation and hyphal development (Argimon et al., 2007). Another gene, *ALS1* encodes cell-surface associated glycoproteins which had shown relatively downregulated during initial biofilm formation (Nailis et al., 2009). Consistently, in this study, an increase in *ALS1* expression was noted after alizarin treatment, suggesting that alizarin might disturb biofilm formation at the initial stage. *ECE1* encodes a protein required for hyphal cell elongation and biofilm formation (Nobile et al., 2006a), and *ECE2* (*HWP1*) is essential for its hyphal development and cell adherence (Nobile et al., 2006b). *HWP1* homolog gene, *RBT1* expression correlates with hyphal formation and biofilm development (Braun et al., 2000). Our data suggest that alizarin and chrysazin inhibits hyphae and biofilm formation by downregulating these hypha-specific genes.

The protein encoded by *EFG1* has a dual role as a transcriptional activator or repressor that controls hyphal morphogenesis. Accordingly, decline of *EFG1* transcriptional level was correlated with the hyphal induction (Stoldt et al., 1997; Tebarth et al., 2003). Our results suggest that increase in the transcriptional level of *EFG1* after alizarin treatment (Figure 7) may be due to a feedback loop that controls *EFG1* expression during the maintenance phase of hyphal morphogenesis. Taken together, our results suggest that alizarin and chrysazin may inhibit cell adhesion, biofilm formation, and hyphal development by regulating the hypha-specific genes.

In the previous studies, we observed *C. albicans* cells readily enter the nematode intestine and kill worms, and that the presence of terpenes and terpenoids such as linalool, camphor, and α-longipinene rescued infected nematodes (Manoharan et al., 2017a,b). In another study, terpenoids like gymnemic acid, and polyphenolic class of compounds such as magnolol and honokiol reduced *C. albicans* colonization in the body of nematodes and prolonged the survival rates (Vediyappan et al., 2013; Sun et al., 2015). Based on the observed higher survival rate of infected nematodes, our results suggest that alizarin and chrysazin effectively reduced *C. albicans* virulence *in vivo* (Figure 8A). Although, a correlation exists between compound toxicities in nematodes and mammals, the use of high concentrations of compounds in man is potentially hazardous (Williams and Dusenbery, 1988; Cole et al., 2004; Sochova et al., 2006). Animal toxicities of alizarin, chrysazin, and fluconazole showed that alizarin and chrysazin are less toxic than fluconazole while they prolonged the survival rates of nematodes (Figure 8). Our results suggest that alizarin and chrysazin could provide safe clinical approach in human system by treating biofilm associated *C. albicans* infections.

## Conclusion

Present study shows alizarin and chrysazin inhibit biofilm formation and hyphal development by *C. albicans*. Accordingly, we suggest alizarin and chrysazin have potential use *in vitro* and *in vivo*, and that they offer potential means of treating candidiasis. We suggest that further *in vivo* experiments using mice model will confirm the potential applicability of alizarin and chrysazin against fluconazole resistant *C. albicans*, which could be alternative for commercially available and toxic antifungal agents.

## Author contributions

RM and JL performed experiments, analyzed data, and wrote the manuscript. J-HL and JL designed the study and YK performed microscopic experiments. All authors have read and approved the final manuscript.

### Conflict of interest statement

The authors declare that the research was conducted in the absence of any commercial or financial relationships that could be construed as a potential conflict of interest.

## Supplementary material

Supplementary Figure 1 Antibiofilm activities of anthraquinone derivatives against mixed biofilms of *C. albicans* and *S. aureus*. The antibiofilm activities of alizarin, chrysazin, purpurin and fluconazole were investigated by co-culturing of *C. albicans* and *S. aureus* strains for 24 h in 96-well polystyrene plates in mixed medium (50% LB and 50% PDB) at 37°C. At least two independent experiments were conducted (6 wells per sample). Error bars indicate standard deviations. ^*^*p* < 0.05 vs. non-treated controls.
