# Supplementary figures and images for: Alizarin and Chrysazin Inhibit Biofilm and Hyphal Formation by Candida albicans
Source: Front Cell Infect Microbiol. 2017 Oct 16;7:447. doi: 10.3389/fcimb.2017.00447 (PMC5650607; doi:10.3389/fcimb.2017.00447)

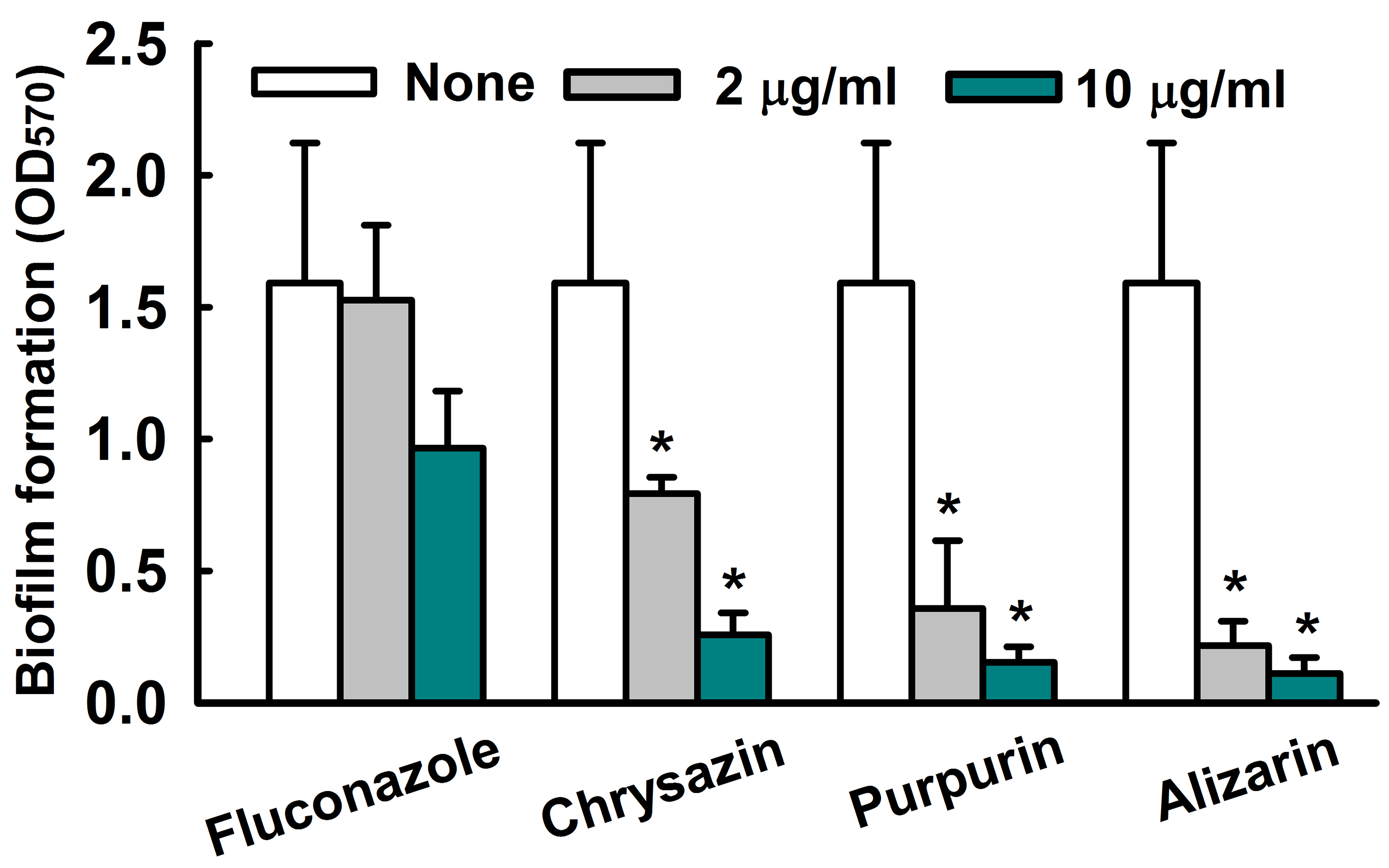

Supplement: Supplementary Figure 1 — Antibiofilm activities of anthraquinone derivatives against mixed biofilms of C. albicans and S. aureus. The antibiofilm activities of alizarin, chrysazin, purpurin and fluconazole were investigated by co-culturing of C. albicans and S. aureus strains for 24 h in 96-well polystyrene plates in mixed medium (50% LB and 50% PDB) at 37°C. At least two independent experiments were conducted (6 wells per sample). Error bars indicate standard deviations. *p < 0.05 vs. non-treated controls. [file Image1.TIF]
