# Supplementary material for: Alizarin and Chrysazin Inhibit Biofilm and Hyphal Formation by Candida albicans
Source: Front Cell Infect Microbiol. 2017 Oct 16;7:447. doi: 10.3389/fcimb.2017.00447 (PMC5650607; doi:10.3389/fcimb.2017.00447)
Supplement: Supplementary file 2 [file Table1.DOCX]

**Supplementary Table 1.** Sequences of the primers used for quantitative RT-PCR

| **Group** | **Gene** | **Function** | **Primer (5’-3’)** | **References** |
| --- | --- | --- | --- | --- |
| House-keeping | *RDN18* | Structural constituent of ribosome/Translation | Forward - AGAAACGGCTACCACATCCCA | (Li et al., 2012) |
|  |  |  | Reverse - CGAATGGGCCCTGTATCGT |  |
| Hypha-specific | *HYR1* | Hyphally regulated protein | Forward - TTGTTTGCGTCATCAAGACTTTG | (Tsang et al., 2012) |
|  |  |  | Reverse - GTCTTCATCAGCAGTAACACAACCA |  |
|  | *EFG1* | Enhanced filamentous growth protein (Positive regulator for *ECE1*, *ECE2* and *ALS3*) | Forward - TATGCCCCAGCAAACAACTG | (Tsang et al., 2012) |
|  |  |  | Reverse - TTGTTGTCCTGCTGTCTGTC |  |
|  | *ALS1* | Agglutinin-like protein 1 | Forward - AGCTGTTGCCAGTGCTTC  Reverse - AATGTGTTGGTTGAAGGTGAG | (Hsu et al., 2013) |
|  | *ALS3* | Agglutinin-like protein 3 | Forward - CAACATCAACCAACCAATCTC  Reverse - TGAATAACAGAACCAGATCCG | (Tsang et al., 2012) |
| Biofilm/  adhesion | *ECE1* | Extent of cell elongation  protein | Forward - CCAGAAATTGTTGCTCGTGTTGCCA | This study |
|  |  |  | Reverse - TCCAGGACGCCATCAAAAACGTTAG |  |
|  | *ECE2 (HWP1)* | Hyphal cell wall protein | Forward - TGGTGCTATTACTATTCCGG | (Sun et al., 2015) |
|  |  |  | Reverse - CAATAATAGCAGCACCGAAG |  |
|  | *RBT1* | HWP1 homolog | Forward- CTGCCATTCAACCATCTGCTAACTCCTCATAC | This study |
|  |  |  | Reverse- CAGCAAGACCAATAATAGCAGCACCATAAGT |  |
|  | *EED1* | Epithelial escape and dissemination | Forward - AGCAACGACTTCCAAAAGGA | (Hsu et al., 2013) |
|  |  |  | Reverse - CGGTTTCTGGTTCGATGATT |  |
